# *Lactiplantibacillus plantarum* LZU-J-Q21 enhanced the functional metabolic profile and bioactivity of *Cistanche deserticola*

**DOI:** 10.1016/j.fochx.2024.101941

**Published:** 2024-10-30

**Authors:** Kangkang Liu, Junxiang Li, Wenting Hao, Jingjing Li, Israr Khan, Yibo Liang, Haijuan Wang, Xiaofeng Li, Chunjiang Zhang

**Affiliations:** a School of Pharmacy, Gansu University of Chinese Medicine, Lanzhou 730101, PR China; b School of Life Sciences, Lanzhou University, Lanzhou 730000, PR China; c Key Laboratory of Cell Activities and Stress Adaptations, Ministry of Education, Lanzhou University, Lanzhou 730000, PR China; d Center for Pharmacovigilance of Gansu Province, Lanzhou 730070, PR China; e Gansu Institute for Drug Control, Lanzhou 730030, PR China

**Keywords:** *Cistanche deserticola*, Submerged fermentation, Anti-fatigue, Metabolic profile

## Abstract

Microbial fermentation is an effective method to enhance the bioavailability of herbs. This study utilized *Lactiplantibacillus plantarum* LZU-J-Q21 to ferment *Cistanche deserticola* and evaluated its metabolic properties and biological activity. Results showed that the contents of total acid and flavone, and the clearance rates of DPPH, ABTS and OH^−^ in fermented *Cistanche deserticola* (FCD) were increased by 142.74 %, 56.45 %, 58.1 %, 62.3 %，51.2 %, compared with non-fermented *Cistanche deserticola* (NFCD). The metabolic profile of FCD had remarkable changes, especially elevated glucose and adenosine (97.31 % and 59.18 %). Further, FCD increased the weight-bearing swimming time of mice by 88.57 %, reduced fatigue markers BUN, BLA, and MDA (18.47 %, 12.92 %, and 15.16 %), and enhanced liver/muscle glycogen and SOD (28.99 %, 28.57 %, and 14.47 %). The investigation into its anti-fatigue mechanism suggested that FCD enhanced GS protein expression by activating PI3K/AKT/GSK3β signaling. These findings suggest that FCD enhances anti-fatigue effects by modifying its metabolic properties and biological activity.

## Highlights

• Fermentation of *Cistanche deserticola* (CD) using LZU-J-Q21.
• Fermentation changed the bioactive compound content and antioxidant activities.
• Metabolic components change, especially those exerting anti-fatigue effects.
• LZU-J-Q21 fermented CD can improve the anti-fatigue effect in mice.

## 1 Introduction

The rapid development of society has resulted in suboptimal health in an increasing number of individuals, with fatigue being a prominent symptom (Pearson et al., 2018). Fatigue may stem from several sources, including overwork, long hours, excessive stress (Griepentrog, Labiner, Gunn, & Rosengart, 2018), and illness (Zhen, Smith, Manji, Schron, & Berry, 2020). A survey conducted in 2021 in six regions in China reported a 67.47 % rate of physiological suboptimal health (Yunlian et al., 2021). In 2023, the World Health Organization (WHO) reported that 6.2 % of the 1.2 million surveyed COVID-19 patients still showed at least one symptom three months post-infection, with fatigue being the most prevalent (Wulf Hanson et al., 2022). Prolonged fatigue can lead to various health issues including insomnia, cognitive impairment, and cancer (Coomans et al., 2019; Ning, Fang, & Zhang, 2023). Although medications like modafinil are often used to treat fatigue, they pose risks of addiction and dependence (Wuo-Silva et al., 2016). Therefore, identifying safe and effective strategies to alleviate fatigue is crucial for improving overall well-being.

With the rise in popularity of complementary and alternative medicine, more people are exploring plant-based foods and traditional remedies as potential solutions (Zou et al., 2016). These options are viewed as safer than chemical drugs and aim to holistically regulate bodily function and overall balance (Shi et al., 2019). For example, research has shown that *sea buckthorn* polysaccharides can significantly enhance anti-fatigue responses in mice (Ni et al., 2013). In addition, verbascoside was shown to enhance mitochondrial function in mouse C_2_C_12_ myoblasts exposed to H_2_O_2_ by increasing mitochondrial spare respiratory capacity (Sciandra et al., 2023). However, the bioavailability of plants consumed directly is often low, and extracting active ingredients typically involves using organic solvents and complex procedures. Therefore, it is essential to utilize economical, safe, and efficient processing methods.

Fermentation is a process that utilizes microorganisms (mainly bacteria) to modify substances in plants and can lead to various new activities under specific environmental conditions (Motarjemi & Nout, 1996). Microbial fermentation can transform macromolecules such as polysaccharides, proteins, and lipids into diverse active molecules that exhibit a broad-spectrum of bioactivities (Ardö, 2006; Septembre-Malaterre, Remize, & Poucheret, 2018). Lactic acid bacterial fermentation is a sustainable and eco-friendly method widely employed in food processing and healthcare. Utilizing *Lactiplantibacillus plantarum* (*L. plantarum*) not only enhances flavors but also offers potential health advantages such as lowering blood pressure and improving blood lipids (Estruch & Lamuela-Raventós, 2023). Therefore, continued research in this area is crucial.

*Cistanche deserticola* (CD), also known as desert ginseng, is primarily found in the dry regions of Northwest China, it primary includes Gansu Province, Qinghai Province, the Inner Mongolia Autonomous Region, and the Xinjiang Uygur Autonomous Region (Xue et al., 2024). This plant has long been highly regarded for its nutritional and medicinal properties (Wang, Zhang, & Xie, 2012), making it a popular ingredient in various food products. This study utilized *L. plantarum*, isolated from ‘JiangShui’, a traditionally fermented food from Gansu Province (Chen et al., 2016), to ferment CD. Our previous studies have shown that *L. plantarum* changes the metabolic profile and bioactivity of herbs, such as fermented Astragalus showing better efficacy in improving ulcerative colitis than non-fermentation (Li et al., 2022). To determine the physical and chemical properties of fermented *Cistanche deserticola* (FCD), experiments were conducted on pH, total acid, total polysaccharides, phenylethanoid glycosides, total flavonoids, and antioxidant activity. Additionally, headspace solid-phase microextraction gas chromatography–mass spectrometry (HS-SPME-GC–MS) and ultra-high performance liquid chromatography-quadrupole/electrostatic field orbitrap high-resolution mass spectrometry (UPLC-Q-Orbitrap-MS) were utilized to carry out metabolic profiling of FCD and non-fermented *Cistanche deserticola* (NFCD). Weight-bearing swim experiments were performed to evaluate the anti-fatigue potential of FCD in mice. Furthermore, the effects of FCD on adverse physiological changes in fatigued mice was studied by assessing fatigue-related parameters (BUN, BLA, liver/muscle glycogen, SOD, and MDA), and expression of related proteins in the PI3K/AKT/GSK3β signaling. This study provides a foundational understanding of the anti-fatigue effects and potential value of FCD for treating fatigue.

## 2 Materials and methods

### 2.1 Materials and reagents

CD was purchased from the Deshengtang Group Co., Ltd. (Lanzhou, China). Strains of *L. plantarum* (LZU-J-Q21, LZU-J-Q25, LZU-J-QA85, LZU-J-TSL6, and LZU-S-ZCJ) were previously isolated from ‘JiangShui’. The strains were deposited at the Guangdong Microbial Culture Collection Center with the following accession numbers: GDMCC 63277, GDMCC 63278, GDMCC 61192, GDMCC 61242, and GDMCC 61402. *Lactiplantibacillus rhamnosus* GG (LGG) (BNCC-185356) was purchased from Beina Chuanglian Biotechnology (Beijing, China). MRS agar and MRS broth were sourced from Qingdao Hope Bio-Technology Co., Ltd. (Shandong, China). DPPH and ABTS were purchased from Shanghai Aladdin Biochemical Technology Co., Ltd. (Shanghai, China). Chromatography-grade methanol and formic acid were purchased from Merck (Darmstadt, Germany). Other reagents and solvents were of analytical purity. BUN, BLA, liver/muscle glycogen, SOD, and MDA kits were purchased from the Nanjing Jiancheng Bioengineering Institute (Nanjing, China). GAPDH and Glycogen synthase (GS) were purchased from Jiangsu Baijia Biotechnology Co., Ltd. (Jiangsu, China), and PI3K, p-PI3K, AKT, p-AKT, GSK3β, and p-GSK3β were purchased from Jiangsu Qinke Biological Research Center Co., Ltd. (Jiangsu, China).

### 2.2 Selection of fermentation strains

A specific quantity of CD was weighed and combined with distilled water at a ratio of 15 mL/g. Then, a 0.2 % complex enzyme solution containing pectinase and cellulase in equal proportions was added to the mixture. The resulting blend was incubated at 60 °C for one hour to create the CD liquid, which was later sterilized by autoclaving at 90 °C for one hour.

The strains were precultured in MRS liquid medium for 18–24 h at an inoculum volume of 2 % (V/V), resulting in a final concentration of approximately 8 Lg CFU/mL. Subsequently, the 2 % V/V volume was added to the CD liquid, and the OD_600_ value was measured using a microplate reader (Bio Tek, USA) every six hours until the fermentation concluded at 36 h.

### 2.3 Optimization of fermentation conditions

The dependent variable in the experiment was the count of viable bacteria found in the sample. A single-factor experimental design was implemented, with variations in fermentation time (6, 12, 18, 24, 30, and 36 h), fermentation temperature (33, 35, 37, 39, and 41 °C), and inoculation amount (1, 2, 3, 4, and 5 %). The viable bacterial count was determined using the agar plate counting method. The results are reported in colony-forming units per milliliter (CFU/mL) and viable counts are expressed as Lg CFU/mL.

### 2.4 Analysis of physicochemical properties

Based on optimal fermentation conditions, *L. plantarum* LZU-J-Q21 was employed to ferment CD (FCD) under the most favorable fermentation conditions. Samples were collected at different time points for further analysis. The non-fermented CD (NFCD) group served as the negative control.

Viable bacteria concentration in the FCD samples during fermentation was assessed using the agar plate counting technique. The pH of the samples was directly measured with a pH meter (Mettler Toledo, S220). The total acid content was quantified according to the national standards of China, GB/T 12456–2021 (Determination of total acid in foods, in Chinese) (PRC, 2021), and expressed as lactic acid equivalents. Total polysaccharides, phenylethanol glycoside, and flavonoid content were determined using the phenol‑sulfuric acid method (Li, Liu, Yang, & Zeng, 2022), UV spectrophotometry (Peng et al., 2022), and the aluminum trichloride method (Albasher, Almeer, Al-Otibi, Al-Kubaisi, & Mahmoud, 2019), respectively, as described in previous studies.

### 2.5 2.4. Antioxidant activity analyses

ABTS radical scavenging activity was assessed using the method described by Huang et al. (2011) with slight modifications. Specifically, 0.1 mL of the sample (supernatant) was combined with 1.9 mL of ABTS· + working solution, incubated in darkness at 25 °C for 30 min, and the absorbance was recorded at 734 nm. The calculation was carried out using Eq. (1).

(1) $$\text{ABTS radical scavenging ability}=\frac{A_{\mathrm{blank}}-A_{\mathrm{sample}}}{A_{\mathrm{blank}}}\times 100\%$$

DPPH radical scavenging activity was assessed using the method described by Palachai, Wattanathorn, Muchimapura, and Thukham-Mee (2019) with slight modifications. Initially, 0.2 mL of the sample (supernatant) was mixed with 2.8 mL of 0.2 mM DPPH solution prepared in absolute ethanol. The mixture was then incubated in the dark at 25 °C for 30 min, and the absorbance was measured at 517 nm. The percentage of DPPH scavenging activity was calculated using Eq. (2).

(2) $$\text{DPPH radical scavenging ability}=\frac{A_{\mathrm{blank}}-A_{\mathrm{sample}}}{A_{\mathrm{blank}}}\times 100\%$$

After 100-fold dilution of the sample supernatant, hydroxyl radical scavenging activity was measured according to the manufacturer's instructions and calculated using Eq. (3).

(3) $$\mathrm{OH}\;\text{radical scavenging ability}=\frac{A_{\mathrm{blank}}-A_{\mathrm{sample}}}{A_{\mathrm{blank}}}\times 100\%$$

### 2.6 Volatile compound analysis

The volatile components were analyzed using HS-SPME-GC–MS following the method outlined by Li et al. (2020). A 1 mL sample with 2 μL of 2-octanol (internal standard solution) (100 μg/mL) was placed into a 20 mL headspace vial. Samples were incubated at 50 °C for 15 min and then mixed. Then, volatile compounds were detected on a DB-wax column (30 m × 0.25 mm × 0.25 μm) using an Agilent 7890B GC–MS system (Agilent, USA) equipped with a LEGO Pegasus BT time-of-flight mass spectrometer. Helium was used as the carrier gas at a flow rate of 1.0 mL/min. The GC oven temperature was initially at 40 °C for 5 min and then increased by 5 °C/min until reaching 220 °C. Subsequently, the temperature was raised to 250 °C at a rate of 20 °C/min and held at that temperature for 2.5 min. The temperature of the inlet, ion source, and quadrupole were set at 260 °C, 230 °C, and 150 °C, respectively. The mass spectrometer was operated in full scan mode between *m*/*z* 20 and 400 with an electron energy of 70 eV. The total analysis cycle time was 50 min. Volatile compounds were identified by comparing the mass spectra with the National Institute of Standards and Technology (NIST) standard library.

### 2.7 Non-volatile compound analysis

Non-volatile metabolites were quantified using a modified protocol (Demurtas et al., 2021). Initially, 2 mL of the sample was mixed with 400 μL of methanol and thoroughly combined. The resulting supernatant was collected, concentrated, and dried. Subsequently, 150 μL of 2-chloro-L-phenylalanine (4 ppm) in a methanol/water (4:1, V/V) solution was used as an internal standard in each sample. The resulting supernatant was filtered through a 0.22 μm membrane and transferred to a detection bottle for LC-MS analysis. In addition, quality control (QC) samples were prepared by mixing an equal volume of supernatant from all samples.

The LC analysis was performed using a Vanquish UHPLC system (Thermo, USA) equipped with an ACQUITY UPLC® HSS T3 column (2.1 × 100 mm, 1.8 μm; Waters, USA). The column was maintained at a constant temperature of 40 °C, with a sample injection volume of 2 μL and a mobile phase flow rate of 0.3 mL/min. Specific parameters are provided in **Supplementary Table S1**. Metabolites were identified using a Q Exactive Focus instrument (Thermo, USA) with an ESI ion source. Mass spectrometry analysis was carried out in both positive and negative modes. Instrument settings included a sheath gas pressure of 40 arb, auxiliary gas flow of 10 arb, spray voltage of 3.50 kV and − 2.50 kV for ESI (+) and ESI (−), respectively, and a capillary temperature of 325 °C.

The identification of non-volatile compounds was achieved by comparing their mass spectra with several databases including HMDB (http://www.hmdb.ca), Mass Bank (http://www.massbank.jp/), and a metabolite database established by Panomix Biomedical Tech Co., Ltd. (Suzhou, China).

### 2.8 Anti-fatigue effect of FCD in mice

#### 2.8.1 Animals and experimental design

The study was conducted following the Guidelines for the Management of Experimental Animals in Gansu Province and the Guidelines for the Management of Experimental Animals in China. All experimental procedures and techniques were approved by the Animal Ethics Committee of the Gansu Institute of Drug Control (approval number: 2023–043). A total of 160 male Kunming mice, aged eight weeks and weighing between 16 and 20 g, were obtained from the Lanzhou Veterinary Research Institute (Lanzhou, China). The mice were housed in animal facilities that met specific pathogen-free (SPF) requirements (room temperature of 20–22 °C, humidity of 45–55 %, 12-h light/dark cycle) with unrestricted access to a standard laboratory diet and water.

After one week of adaptive feeding, the mice were randomly divided into four main groups for subsequent experiments. Each main group was further divided into five groups, each consisting of eight mice (*n* = 8). The groups were organized as follows: the control group (referred to as the ‘C group’), the non-fermented group (referred to as the ‘N group’), and three fermented groups referred to as the low, medium, and high (abbreviated as the ‘L,’ ‘M,’ and ‘H' groups, respectively). The mice in the C group received 0.2 mL of normal saline daily. The N and L groups were given a daily dosage of 1.21 g/kg of NFCD and FCD, respectively. The M group received 6.05 g/kg of FCD daily, while the H group received 12.1 g/kg FCD daily. The experiment lasted a total of 30 days.

#### 2.8.2 Weight-bearing swimming experiment

For Experimental Set 1, the mice were loaded with lead skin equivalent to 5 % of their body weight at the base of their tails. Thirty minutes after the last administration, the mice were placed in a swimming box with a water depth of 40 cm and a temperature of 25 °C ± 1.0 °C. The time from the beginning of swimming to the death of the mouse, known as the weight-bearing swimming time, was recorded.

#### 2.8.3 Biochemical indicator detection

For Experimental Set 2, the mice were subjected to a swimming test in water maintained at a temperature of 30 °C for 90 min. No additional weight was added during the test. The test was conducted thirty minutes after the final oral administration. Following a one-hour rest period, the eyeball was removed, and a 0.5 mL blood sample was collected without the use of an anticoagulant. The collected sample was then stored in a refrigerator at 4 °C for three hours. Subsequently, the sample was centrifuged at a speed of approximately 2000 rpm for 15 min. The upper serum obtained from the centrifuged sample was utilized to measure the levels of blood urea nitrogen (BUN).

For Experimental Set 3, mice were orally administered samples. After a 30-min interval, the mice underwent a 10-min water-based swimming activity at 30 °C, without any additional burdens. The levels of blood lactate (BLA) were measured at three different time intervals: t _−10_ min (before swimming), t _0_ min (immediately after swimming), and t _20_ min (20 min after swimming). The area under the curve (AUC) was determined utilizing Eq. (4);

(4) $$\mathrm{AUC}=5\times \left({\mathrm{BLA}}_{t-10}+3\times {\mathrm{BLA}}_{t\;0}+2\times {\mathrm{BLA}}_{t\;10}\right)$$

For Experimental Set 4, blood samples were collected 30 min after the final oral administration. Subsequently, the animals were euthanized using cervical dislocation, and the liver and left hindleg skeletal muscles were extracted. The extracted tissues were rinsed with cold saline and dried using filter paper. Glycogen levels in both the liver and muscle were measured. The blood samples were stored at 4 °C for 3 h, followed by centrifugation at a speed of 2000 r/min for 25 min to obtain serum. The levels of SOD and MDA were then determined in the obtained serum.

### 2.9 Histopathological analysis

For Experimental Set 1, post-mortem dissection of mice yielded the left kidney, liver, and left hindleg skeletal muscles. The harvested tissues were fixed in a 4 % paraformaldehyde solution and decalcified in a dedicated solution for 72 h. Subsequently, paraffin-embedded sections were prepared and stained with Hematoxylin-Eosin (HE) using established protocols. Histological analysis was conducted using a panoramic slide scanner to acquire detailed images of the tissue sections.

### 2.10 Western blot analysis

In experimental set 4, proteins were extracted from liver tissue using RIPA lysis buffer, following the established protocol. The concentration of the extracted proteins was determined using the BCA protein assay reagent. Subsequently, the membrane was preincubated with a 5 % solution of skimmed milk powder for one hour, followed by overnight incubation at 4 °C with the primary antibody. After this, the membrane underwent five washes with TBST, each lasting five minutes. This was followed by a 1.5-h incubation at 37 °C with the secondary antibody, which was also succeeded by five washes with TBST, each lasting five minutes. Ultimately, the membrane was analyzed using an ECL detection reagent, and the resulting bands were captured by a gel documentation system. Quantitative analysis of the Western blot bands was performed using ImageJ software.

### 2.11 Statistical analysis

All experiments were repeated at least three times, and the data are expressed as the mean ± standard deviation. Statistical analyses were performed using GraphPad Prism 9.5 (GraphPad Software, Inc., USA). One-way analysis of variance (ANOVA) was used to determine statistical significance. The threshold for statistical significance was set at *p* < 0.05. For conducting principal component analysis (PCA), partial least squares-discriminant analysis (PLS-DA), and orthogonal partial least squares-discriminant analysis (OPLS-DA), we employed using the R XCMS package.

## 3 Results

### 3.1 CD fermentation and optimization of conditions

**Fig. 1:**
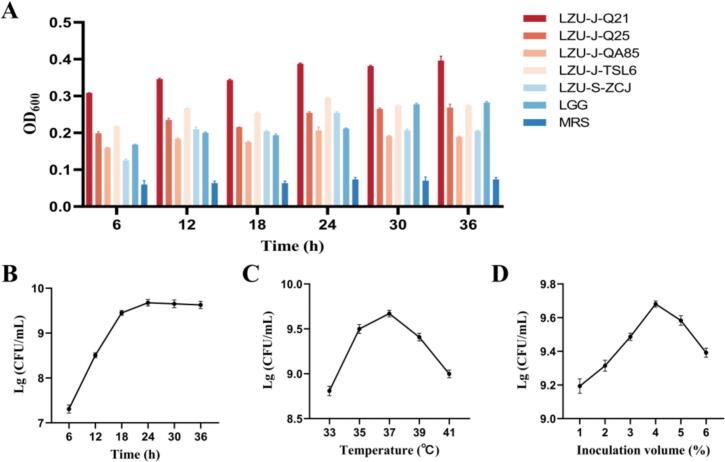
Fermentation of CD for 36 h using different strains (A). The impact of fermentation time (B), temperature (C), and amount of inoculation (D) on the quantity of live bacteria present in the CD fermentation.

Various strains were utilized to ferment CD. Results showed that *L. plantarum* LZU-J-Q21 exhibited consistently higher OD_600_ levels throughout the fermentation process compared to other strains (Fig. 1A). Consequently, *L. plantarum* LZU-J-Q21 was selected for further optimization of the fermentation conditions. Subsequent findings (Fig. 1B–D) revealed that the optimal fermentation parameters included a 24-h fermentation time, a temperature of 37 °C, and an inoculation amount of 4 % V/V.

### 3.2 Physical and chemical properties analysis of FCD

**Fig. 2:**
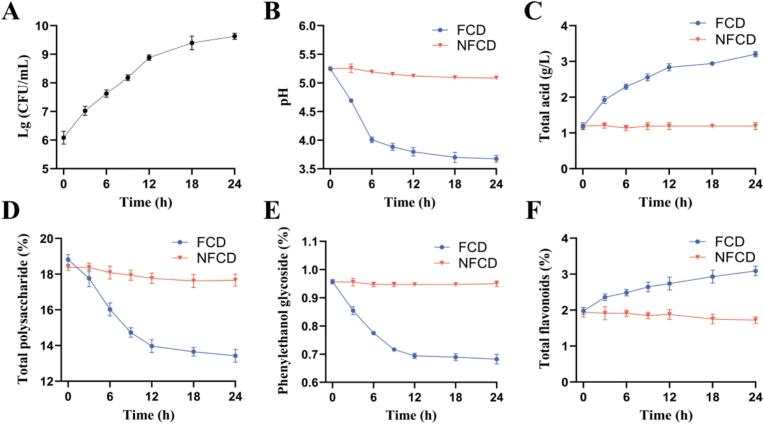
Changes in viable cell number and physicochemical properties of CD during *L. plantarum* LZU-J-Q21 fermentation A. Viable cell number. B. pH. C. Total acid content. D. Total polysaccharide content. E. Phenylethanol glycoside content. F. Total flavonoid content.

To investigate changes in FCD over time, several indicators were assessed. In the absence of additional nutrients, *L. plantarum* LZU-J-Q21 exhibited rapid growth during the initial 12 h of the fermentation cycle, culminating in a final concentration of 9.63 Lg CFU/mL (Fig. 2A). The pH level decreased from 5.24 to 3.80 within the first 12 h, while the total acid content exhibited an opposite trend, rising from 1.17 g/L to 2.84 g/L (Fig. 2B–C). The primary active components in CD, total polysaccharides and phenylethanol glycosides, notably decreased within the initial 12 h, with reductions of 31.55 % and 39.23 %, respectively, by the end of fermentation (Fig. 2D–E). In contrast, the total flavonoid content demonstrated a consistent increase throughout the fermentation process, ultimately rising by 56.45 % (Fig. 2F). These results suggest that the physical and chemical properties of CD undergo significant changes during fermentation, particularly within the initial 12 h.

### 3.3 Antioxidant activity

**Fig. 3:**
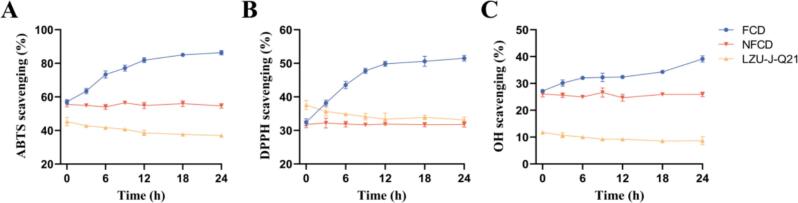
*In vitro* antioxidant activity during *L. plantarum* LZU-J-Q21 fermentation of CD A. ABTS radical scavenging activity. B. DPPH radical scavenging activity. C. OH radical scavenging activity.

Three methods were employed to comprehensively assess the antioxidant properties of FCD; results are depicted in Fig. 3A–C. The free radical scavenging capacity of FCD demonstrated a consistent upward trend with fermentation time. By the end of fermentation, ABTS, DPPH, and OH free radical scavenging activities had risen by 58.1 %, 62.3 %, and 51.2 %, respectively, compared to the NFCD group.

### 3.4 Volatile compound analysis

**Fig. 4:**
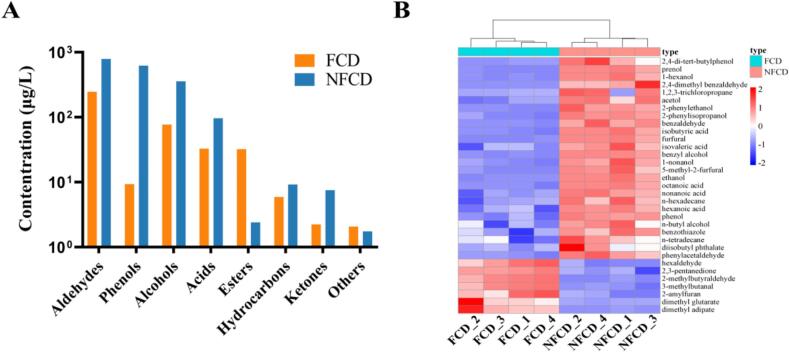
Volatile compound analysis A. Content of different classes of volatile metabolites. B. Heatmap of the volatile compounds.

This study utilized HS-SPME-GC–MS to analyze changes in key volatile compounds before and after fermentation. Total ion chromatograms can be found in **Supplementary Fig. S1**. A total of 33 different volatile metabolites were identified using *t-*test screening (*P* ≤ 0.05) and VIP scoreing based on the PLS-DA model. The metabolites were categorized as eight aldehydes, two phenolics, eight alcohols, five acids, three esters, three hydrocarbons, two ketones, and two other compounds (Fig. 4A). A cluster heat-map was generated to facilitate a comparison of changes in each volatile metabolite (Fig. 4B). The results showed that compared to the NFCD group, the FCD group exhibited increased levels of seven volatile substances: dimethyl adipate, dimethyl glutarate, 2-pentylfuran, isovaleraldehyde, 2-methylbutyraldehyde, 2,3-pentanedione, and n-hexanal. The top five compounds that exhibited the largest decrease in the FCD group were: 2,4-di-tert-butylphenol, 3-methyl-2-buten-1-ol, n-hexanol, 2,4-dimethylbenzaldehyde, and 1,2,3-trichloropropane. The corresponding mass spectrograms are presented in **Supplementary Fig. S2A-E**.

### 3.5 Non-volatile compound analysis

Evaluation of the accuracy and reproducibility of metabolite detection was performed using superimposed display analysis of the total ion current (TIC) data acquired via mass spectrometry. The TIC curves exhibited significant overlap (**Supplementary Fig. S3A—B**), demonstrating consistent signal stability across different time points for the same sample. Quality control (QC) samples were incorporated to ensure stable and accurate data collection. The results showed a clear clustering trend among the QC samples, with values falling within a 95 % confidence interval (**Supplementary Fig. S3C—D**), indicating good repeatability. Additionally, a principal component analysis (PCA) was conducted on two distinct sample groups, FCD and NFCD, which resulted in visually distinct clusters (**Supplementary Fig. S3E—F**). These findings indicate that fermentation significantly influenced CD metabolites. Overall, these results strongly support the reliability and reproducibility of the profiling data.

**Fig. 5:**
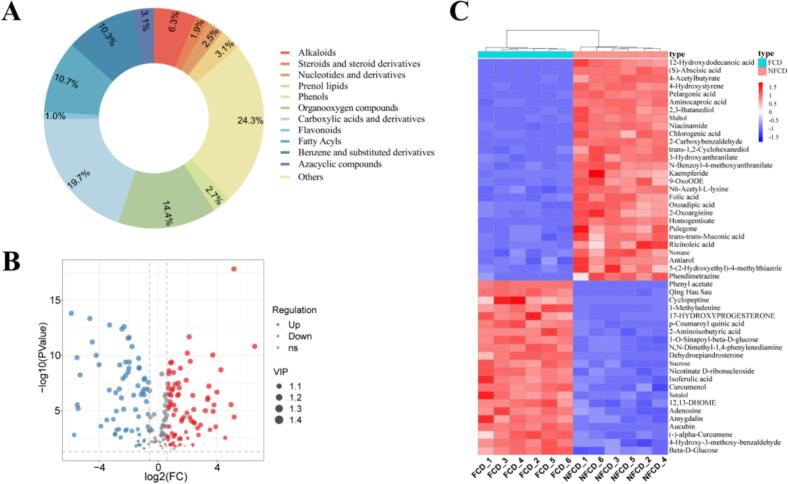
Non-volatile compound analysis A. Content of different classes of non-volatile metabolites. B. Volcano plot of the statistical data. C. Heatmap of differentially abundant metabolite expression between samples.

We detected a total of 16,143 non-volatile metabolite features in ESI+ mode and 8586 non-volatile metabolite features in ESI- mode. To identify the differentially abundant metabolites, we conducted a first-level difference analysis and filtered the results using a preset *P* value and VIP threshold. Manual screening revealed 478 non-volatile metabolites in the FCD and NFCD samples. Among these, 302 metabolites were identified in ESI+ mode, and 176 were identified in ESI- mode. Fig. 5A illustrates the different classes of non-volatile metabolites, including alkaloids, steroids, and steroid derivatives, nucleotides and derivatives, prenol lipids, phenols, organooxygen compounds, carboxylic acids and derivatives, flavonoids, fatty acyls, benzene, substituted derivatives, azacyclic compounds, and other metabolites. The volcano plot (Fig. 5B**)** visually depicts the distribution and trends of differentially abundant metabolites between the two sample groups. The analysis utilized color coding: red for significantly upregulated metabolites, blue for downregulated metabolites ones, and gray for metabolites with no significant difference. Among the 191 significantly altered metabolites (VIP ≥ 1), 110 showed increases while 81 decreased. The mass spectra of five energy metabolism-related compounds are presented in **Supplementary Fig. S4A–E**. To better comprehend the comparative changes in metabolites between the FCD and NFCD groups, we conducted hierarchical cluster analysis-heatmaps on the top 50 metabolites based on the *P* value. These metabolites were categorized into two clusters (Fig. 5C**)**, aligning with the PCA results.

### 3.6 KEGG analysis

**Fig. 6:**
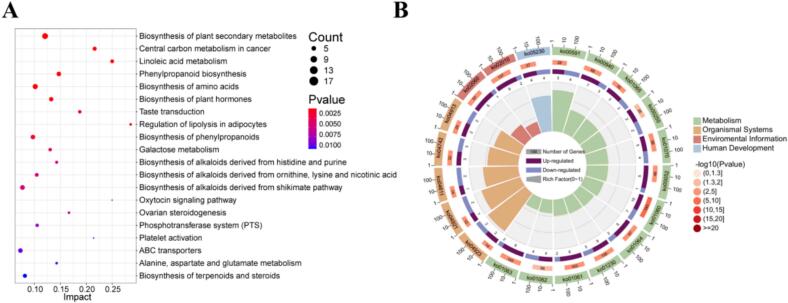
KEGG enrichment analysis A. Bubble chart of the top 20 enriched KEGG pathways. B. KEGG enrichment circle diagram (from the outside to the inside, the first circle represents the top 20 enriched pathways, and the number outside the circle is the coordinate ruler of the number of metabolites; the second circle represents the number and Q value of background metabolites in the pathway; the more genes, the longer the bar; the third circle represents a bar chart of the ratio of up-regulated and down-regulated metabolites, with dark purple representing the ratio of up-regulated metabolites and light purple representing the ratio of down-regulated metabolites; the fourth circle represents the value of the Rich Factor in each pathway). (For interpretation of the references to color in this figure legend, the reader is referred to the web version of this article.)

The differentially abundant metabolites were analyzed using the MetaboAnalyst tool to identify relevant pathways impacted by fermentation. Enrichment analysis was conducted using the hypergeometric test, and the topology of the pathways was assessed using degree centrality as a parameter. The top 20 pathways identified in the KEGG enrichment analysis (arranged by *P* value) are presenetd in Fig. 6A. The findings indicate that fermentation primarily impacted the biosynthesis of phenylpropanoids, the biosynthesis of alkaloids derived from the shikimate pathway, ABC transporters, phenylpropanoid biosynthesis, galactose metabolism, and the phosphotransferase system (PTS). The OmicShare tool (https://www.omicsshare.com/tools/) was utilized to present these pathways more clearly. As illustrated in Fig. 6B, these pathways can be broadly categorized into four main groups: Metabolism, Organismal Systems, Environmental Information Processing, and Human Diseases. Given that fermentation is a biochemical process of microbial metabolism, its effects were primarily observed in metabolism-related pathways, particularly those associated with amino acids.

### 3.7 Anti-fatigue effect of FCD in mice

#### 3.7.1 Phenotypic indicators

**Fig. 7:**
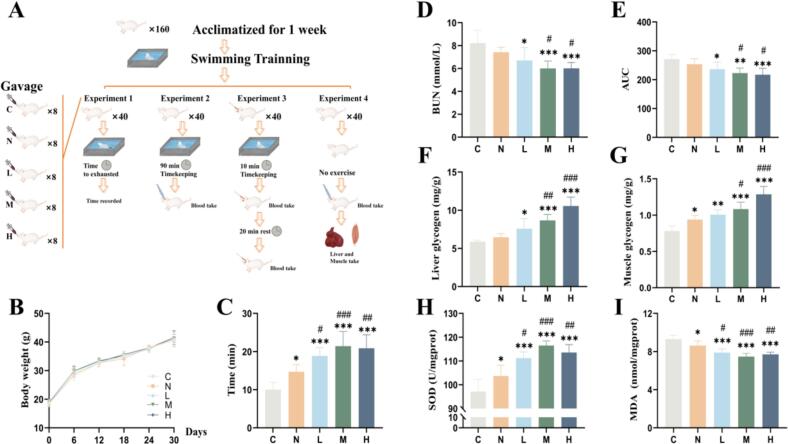
Schematic of the experiment testing anti-fatigue effect of FCD A. Animal experimental design. B. Changes in animal body weight. C. Weight-bearing swimming time. D. Blood urea nitrogen. E. Area under the curve of blood lactate acid. F. Liver glycogen concentration. G. Muscle glycogen concentration. H. SOD content. I. MDA content. The data are expressed as the mean ± SD (*n* = 8). *P < 0.05, **P < 0.01, ***P < 0.001 compared to group C; #*P* < 0.05, ##*P* < 0.01, ###*P* < 0.001 compared to group N.

The schematic diagram of the animal experi'mental procedure is presented in Fig. 7A. Throughout the experimental period, all groups of mice maintained normal general condition, active behavior, and good mental state. Weight gain patterns were consistent across all groups with no abnormal changes observed (Fig. 7B). The weight-bearing swimming test showed that all treatment groups swam significantly longer than the control group (Fig. 7C). Specifically, swimming times for groups N, L, M, and H were 45.14 %, 88.57 %, 114.29 %, and 108.57 % longer than the control group, respectively. Furthermore, groups L, M, and H swam 28.16 %, 45.63 %, and 41.75 % longer than group N, respectively.

#### 3.7.2 Biochemical indicators

Group N had lower BUN levels than group C, but this difference was not statistically significant. In contrast, groups L, M, and H showed significantly lower BUN levels, with reductions of 18.47 %, 26.93 %, and 26.77 %, respectively, compared to group C (*P* < 0.05) (Fig. 7D). In addition, analysis of data from **Supplementary Table S2** indicated that before swimming, mice in all groups had similar BLA levels. After the swimming exercise, BLA levels increased by 56.93 %, 46.19 %, 46.41 %, 43.97 %, and 39.41 % in groups C, N, L, M, and H, respectively. Following a 20-min rest period, reductions of 10.37 %, 7.66 %, 20.05 %, 28.97 %, and 24.31 % were observed in the respective groups. Group L also showed greater effectiveness than group N in reducing the AUC (Fig. 7E). The liver glycogen for groups N, L, M, and H were 6.46 mg/g, 7.57 mg/g, 8.67 mg/g, and 10.56 mg/g, respectively, which is 10.10 %, 28.99 %, 47.60 %, and 79.79 % higher than the control group (5.87 mg/g), respectively. Similarly, the muscle glycogen for groups N, L, M, and H were 0.94 mg/g, 1.00 mg/g, 1.08 mg/g, and 1.29 mg/g, respectively, which is 20.10 %, 28.59 %, 38.43 %, and 64.50 % higher than the control group (0.78 mg/g), respectively. Although group N showed improvement compared to group C, these levels remained lower than those of group L (Fig. 7F–G). A similar trend was observed in the mitigation of oxidative stress, with group N displaying decreased MDA levels and elevated SOD levels. However, these effects were less pronounced than those in groups L, M, and H^,^ (*P* < 0.05) (Fig. 7H–I).

#### 3.7.3 Histopathological analysis

**Fig. 8:**
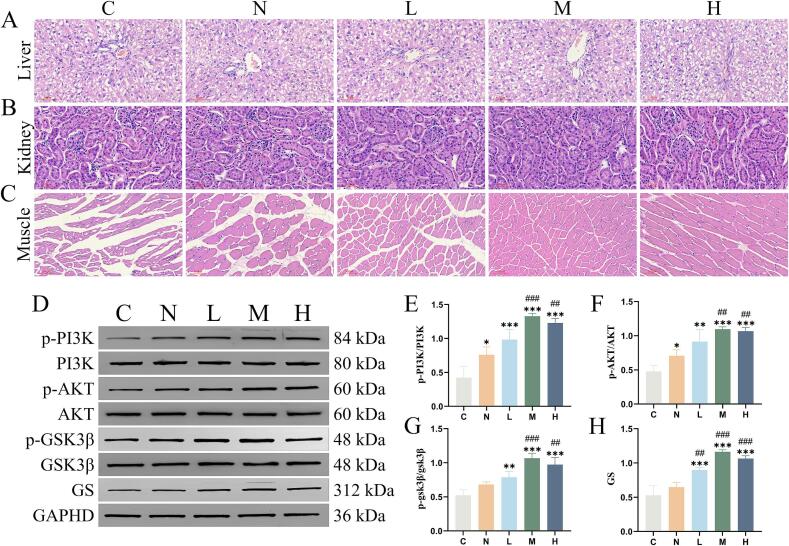
Histopathological sections of the major metabolic organs in mice. Liver (A), Kidney (B), and Muscle (C), (liver and kidney magnification × 200; scale bar = 60 μm; muscle magnification × 100; scale bar = 100 μm). Western blotting detects the expression of PI3K/AKT/GSK3β pathway-related proteins. Western blot data show expression levels of p-PI3K, PI3K, p-AKT, AKT, p-GSK3β, GSK3β,and GS (D). The protein levels shown in (D) are further detailed in (E-H). Data are expressed as mean ± SD (*n* = 3). *P < 0.05, **P < 0.01, ***P < 0.001 compared to group C; ##*P* < 0.01, ###*P* < 0.001 compared to group N.

Fig. 8A-C reveals slight lymphocyte infiltration in the liver across all groups, with the M group showing notably less infiltration. All groups exhibited minor inflammatory cell infiltration in kidney tissue. However, the L, M, and H groups showed a reduction in inflammatory cells compared to the C and N groups. In the C group, skeletal muscle cells appeared shrunken and displayed significant cracks between them. Conversely, skeletal muscle morphology in the N, L, H, and M groups gradually normalized, with the M group showing a particularly flat and dense muscle structure.

#### 3.7.4 Regulatory effect on PI3K/AKT/GSK3β signaling and GS protein expression

In group C, levels of phosphorylated PI3K (p-PI3K), phosphorylated AKT (p-AKT), phosphorylated GSK3β (p-GSK3β), and glycogen synthase (GS) proteins were low. In contrast, the relative expression levels of p-PI3K/PI3K, p-AKT/AKT, p-GSK3β/GSK3β, and GS proteins in the liver tissue of mice from groups N, L, M, and H exhibited increase. Nevertheless, the expression levels of these proteins in group N were significantly lower than those in groups M and H (P < 0.05) (Fig. 8D-H). The original western blotting data are available in the **Supplementary Materials**.

## 4 Discussion

Many studies have shown that different bacterial strains can have diverse impacts on fermentation results, depending on the substrate used. For example, research by Li et al. (2024) demonstrated that distinct bacterial strains displayed varying antioxidant activities during kiwifruit fermentation. Similarly, the fermentation of melon juice with different strains produced diverse effects on volatile compounds (Wang et al., 2023). A straightforward method for selecting suitable fermentation strains is measuring absorbance at OD_600_, and LGG is commonly used in the realm of fermented foods due to its exceptional fermentation capabilities. However, our research indicates that *L. plantarum* LZU-J-Q21 has superior fermentation capability compared to LGG with regard to CD fermentation. In certain cases, alterations in fermentation conditions may not substantially affect OD_600_ measurements. Therefore, employing viable bacterial counts may yield more accurate results when optimizing fermentation parameters.

The primary metabolites produced by *L. plantarum* are organic acids, especially lactic acid, which significantly affect both pH and total acid levels. Previous research (Giri, Sen, Saha, Sukumaran, & Park, 2018) has shown that rice fermented beverages prepared with *L. plantarum* L7 resulted in a decrease in pH and an increase in organic acid content, corroborating our results. Moreover, changes in metabolites can impact pH levels. For instance, Su et al. (2016) showed that verbascoside can be hydrolyzed to from caffeic acid. As verbascoside is a main active component of CD, this may also lead to a decrease in pH. CD contains polysaccharides and phenylethanoid glycosides, which are crucial for its biological functions. During fermentation, *L. plantarum* LZU-J-Q21 utilizes these compounds as a carbon source, leading to a decrease in their levels. This observation aligns with a study by Shi. Wang, and Li (2023) involving *L. plantarum* JHT78 fermentation of watermelon juice, which resulted in a reduction in polysaccharide content from 63.80 mg/mL to 26.31 mg/mL within 12 h. It is important to note that changes in polysaccharide content can vary based on the specific substrate and strain used. For instance, Tang et al. (2023) presented findings that contrasted with those mentioned earlier. Their study showed that fermentation of Dendrobium officinale with *L. plantarum* CCFM8661 led to an increase in total polysaccharide content to 1.40 g/L. This difference between the two studies may be due to changes in polysaccharide structure or the production of exopolysaccharides. The observed increase in total flavonoids may have been due to the microbial breakdown of flavonoid glycosides into aglycones.

Metabolites generated during fermentation can influence antioxidant activity both directly and indirectly. Our results showed a consistent correlation between changes in FCD antioxidant activity, the increase in viable bacteria count, and the rise in total flavonoid levels. This suggests that alterations in antioxidant capacity are primarily driven by microbial fermentation, as evidenced by the stable antioxidant activity in the NFCD group. A related study (Zhang et al., 2023) found that fermenting Gynostemma pentaphyllum leaves with *L. plantarum* ATCC 8014 and SWFU D16 enhanced antioxidant activity, correlating it with increased flavonoid and polyphenol levels. Furthermore, fermentation facilitates the breakdown of plant cell walls, leading to the release or synthesis of added antioxidant compounds (Tao, Feng, Sheng, & Song, 2022).

Odor significantly influences the sensory qualities of food and impacts consumer acceptance. Although plant-based health foods are increasingly popular, their development can be inhibited by unpleasant odors. For instance, garlic is well-known for its anti-cancer and antibacterial effects, primarily due to allicin, a key bioactive compound (Zhang et al., 2020). However, allicin is associated with a strong pungent odor that is off-putting to many individuals (Borlinghaus, Albrecht, Gruhlke, Nwachukwu, & Slusarenko, 2014). A growing trend in to mitigate odor issues in these foods is the application of fermentation. Previous studies (Yan et al., 2022) have demonstrated that fermenting Shenheling Extract with *Lactobacillus fermentum* grx08 effectively reduced specific compounds to improve its odor profile.

Xuyang et al. (2023) revealed that the main volatile elements in CD are aldehydes, alcohols, and ester compounds. These fingdings align with our research results. The decrease in aldehydes may be ascribed to their transformation in acidic environments, where alcohols and acids react to form esters. Moreover, the decline in phenolic compounds is likely linked to oxidation and condensation reactions that take place during fermentation. The decline in overall volatile compound levels might be impacted by the generation of higher alcohols, the oxidation of specific volatile compounds, and sterilization procedures. Notably, the relative content of 2,4-dimethylbenzaldehyde, known for its bitter almond flavor, decreased by 89.6 % after fermentation. These results suggest that the fermentation process using *L. plantarum* LZU-J-Q21 significantly affected the volatile components in CD.

Previous studies have shown that ferulic acid can be converted into isoferulic acid during fermentation (Adeboye, Bettiga, Aldaeus, Larsson, & Olsson, 2015). Similarly, in this study, we observed a decrease in the relative levels of ferulic acid alongside an increase in the isoferulic acid. Isoferulic acid, a derivative of cinnamic acid, has been shown to have antidiabetic activity through the inhibition of the sucrase enzyme (Adisakwattana, Chantarasinlapin, Thammarat, & Yibchok-Anun, 2009). This inhibition reduces the breakdown and conversion of sugar into glucose within the body. Therefore, higher levels of isoferulic acid are associated with increased sucrose content. Furthermore, we detected an increase in the levels of adenosine, an important intermediary in the synthesis of adenosine triphosphate, as well as glucose, the primary source of energy for the human body. This indicates that compared with NFCD, FCD can supply more energy-rich substances to the human body. Recent studies also indicate that CD hepls alleviate fatigue by increasing glycogen levels. Results of this study showed that fermentation can increase the amount of energy-rich substances in CD, thus supporting the superior potential anti-fatigue properties of FCD.

We performed KEGG analysis to better understand the impact of fermentation on the metabolic pathways associated with CD. Phenylpropanoids are secondary metabolites synthesized via the shikimate pathway from aromatic amino acids such as phenylalanine and tyrosine. In plants, these compounds play vital roles in maintaining structural integrity, providing defense, and facilitating cell communication, and have been shown to exert antioxidant and anti-fatigue properties in various studies (Chen et al., 2016). The enzyme responsible for phenylalanine deamination is phenylalanine ammonia-lyase (PAL), which converts phenylalanine to trans-cinnamic acid (Silva et al., 2021). Then, this acid can be hydroxylated to generate phenylpropanoid acids (C6-C3) such as coumaric acid. These compounds serve as direct precursors for the synthesis of lignin and flavonoids. Hence, the increase in flavonoid content observed in our study may be attributed to these compounds.

Galactose metabolism, a biochemical process, facilitates the conversion of galactose into glucose for energy production. One significant outcome of this process is the conversion of stachyose to mannotriose and sucrose through the actions of β-fructofuranosidase (INV) and α-galactosidase (GLA). Notably, this metabolic pathway likely accounts for the significant increase in sucrose levels observed in the FCD group during our study. Sucrose has a high glycemic index (GI) and is rapidly absorbed, making it an excellent source for quick energy replenishment (Qin et al., 2017). In addition, ATP-binding cassette (ABC) transporters play a crucial physiological role by utilizing the energy derived from ATP hydrolysis to facilitate the transfer of substances across membranes (Davidson & Maloney, 2007). This specific protein enables efficient cellular uptake of fermentation substances with a high energy content such as sucrose, α-glucose, and adenosine.

This study found that antioxidant capacity and levels of energy-supplying substances, namely adenosine and beta-d-glucose, increased following the fermentation of CD. Subsequently, animal experiments were conducted to investigate the effect of FCD on fatigue in mice and to delineate the underlying mechanisms. Fatigue in mice can be evaluated by measuring the duration of weight-loaded swimming time (Sun, Yang, Zhao, Bai, & Guo, 2018). Interestingly, research by Li et al. (2022) indicated that treatment with fermented Black Ginseng also led to an increased in weight-loaded swimming time in mice. Our results showed that compared with group C, both NFCD and FCD exhibited significant anti-fatigue effects. In addition, the FCD group demonstrated a greater anti-fatigue effect compared to the NFCD group.

Results of the current study showed that ingestion of FCD decreased BUN and MDA levels in mice while increasing liver and muscle glycogen, as well as SOD content. Interestingly, one study (Cai et al., 2021) discovered that Ganoderma lucidum polysaccharide produced an anti-fatigue effect in mice by reducing MDA, BUN, and BLA levels while increasing SOD levels. These results align with the findings from the current study. This is important because BUN is a key indicator of fatigue level (Zhao et al., 2015), and increased levels signify a diminished capacity to handle stress. In addition, lactic acid is generated during glycolysis. Excess accumulation of lactic acid results in a decrease in pH, which hampers nerve impulse transmission at the neuromuscular junction and leads to muscle soreness and fatigue (Street, Bangsbo, & Juel, 2001). Glycogen, the primary form of glucose storage in the body, directly influences endurance during physical activity (Namma-Motonaga et al., 2022). Oxidative stress reflects an imbalance between oxidation and antioxidants levels in the body. Blood vessels play a crucial role in delivering oxygen and nutrients to all tissues and organs, and elevated levels of free radicals can impede blood vessel dilation, accelerating fatigue (Patsch et al., 2015).

High-intensity exercise increases the body's energy requirements, resulting in greater production of reactive oxygen species (ROS) by mitochondria. Elevated ROS levels can cause oxidative stress in metabolic organs like the liver and kidneys, potentially leading to muscle cell damage (Huang et al., 2018). Supplementing with exogenous antioxidants can mitigate the damage caused by exercise.Our H&E staining findings suggest that FCD acts as an exogenous antioxidant, diminishing exercise-related damage to important metabolic organs. This supports previous observations of FCD's high antioxidant capacity *in vitro* and within liver tissue. The PI3K/AKT/GSK3β signaling pathway is crucial for insulin-mediated glycogen synthesis. GS, a key enzyme in regulating glycogen synthesis, is located at the downstream end of this signaling pathway (Luo et al., 2020). PI3K activates AKT, which subsequently facilitates the phosphorylation of GSK3β. This phosphorylation alleviates the inhibitory effect of GSK3β on GS, thereby enhancing glycogen synthesis. In this study, we investigated the anti-fatigue mechanisms of FCD by quantifying the expression levels of p-PI3K, PI3K, p-AKT, AKT, p-GSK3β, GSK3β, and GS proteins in the livers of mice. Our results indicate that FCD may enhance the phosphorylation of PI3K, AKT, and GSK3β proteins, leading to increased GS protein expression, promoting glycogen synthesis and providing anti-fatigue effects. Compared to NFCD, FCD demonstrates a stronger activation of these signaling pathways, thereby improving anti-fatigue efficacy. In summary, the current results showed that the fermentation process significantly enhanced the anti-fatigue effect of FCD compared to NFCD.

## 5 Conclusions

In conclusion, the experimental results demonstrated that *L. plantarum* LZU-J-Q21 altered the bioactive substance content in CD during fermentation. Additionally, FCD exhibited significant *in vitro* antioxidant properties and contained greater levels of energy-supplying substances comparted to CD. Experiments in mice indicated that FCD effectively decreased BUN, BLA, and MDA levels while increasing liver/muscle glycogen and SOD levels. This effect may be linked to the activation of the PI3K/AKT/GSK3β signaling, which increases GS protein expression and produces a notable anti-fatigue effect. These findings underscore the potential of FCD to increase resistance to fatigue and lay a robust theoretical foundation for its application as a functional food.

## CRediT authorship contribution statement

**Kangkang Liu:** Writing – original draft, Methodology, Investigation, Formal analysis, Data curation. **Junxiang Li:** Writing – original draft, Methodology, Investigation, Formal analysis. **Wenting Hao:** Validation, Software. **Jingjing Li:** Writing – review & editing, Validation. **Israr Khan:** Writing – review & editing. **Yibo Liang:** Investigation. **Haijuan Wang:** Validation, Methodology. **Xiaofeng Li:** Project administration, Conceptualization. **Chunjiang Zhang:** Writing – review & editing, Supervision, Project administration, Funding acquisition, Conceptualization.

## Declaration of competing interest

The authors declare that they have no known competing financial interests or personal relationships that could have appeared to influence the work reported in this paper.

## Data Availability

Data will be made available on request.

## Appendix A Supplementary data

Supplementary material 1 Image 1

Supplementary material 2 Image 2
