# Supplementary material for: Lactiplantibacillus plantarum LZU-J-Q21 enhanced the functional metabolic profile and bioactivity of Cistanche deserticola
Source: Food Chem X. 2024 Oct 30;24:101941. doi: 10.1016/j.fochx.2024.101941 (PMC11577131; doi:10.1016/j.fochx.2024.101941)
Supplement: Supplementary file 1 — Supplementary material 1 [file mmc1.docx]

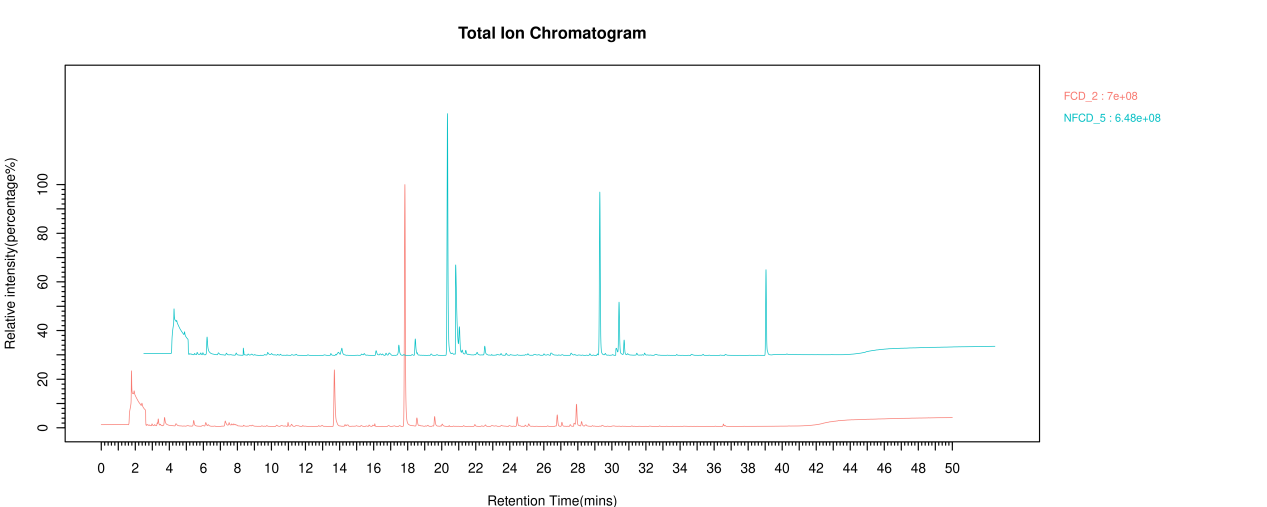


**Supplementary Figure S1:** The total ion chromatogram (TIC) of FCD and NFCD.The abscissa is the retention time of the metabolites detected.


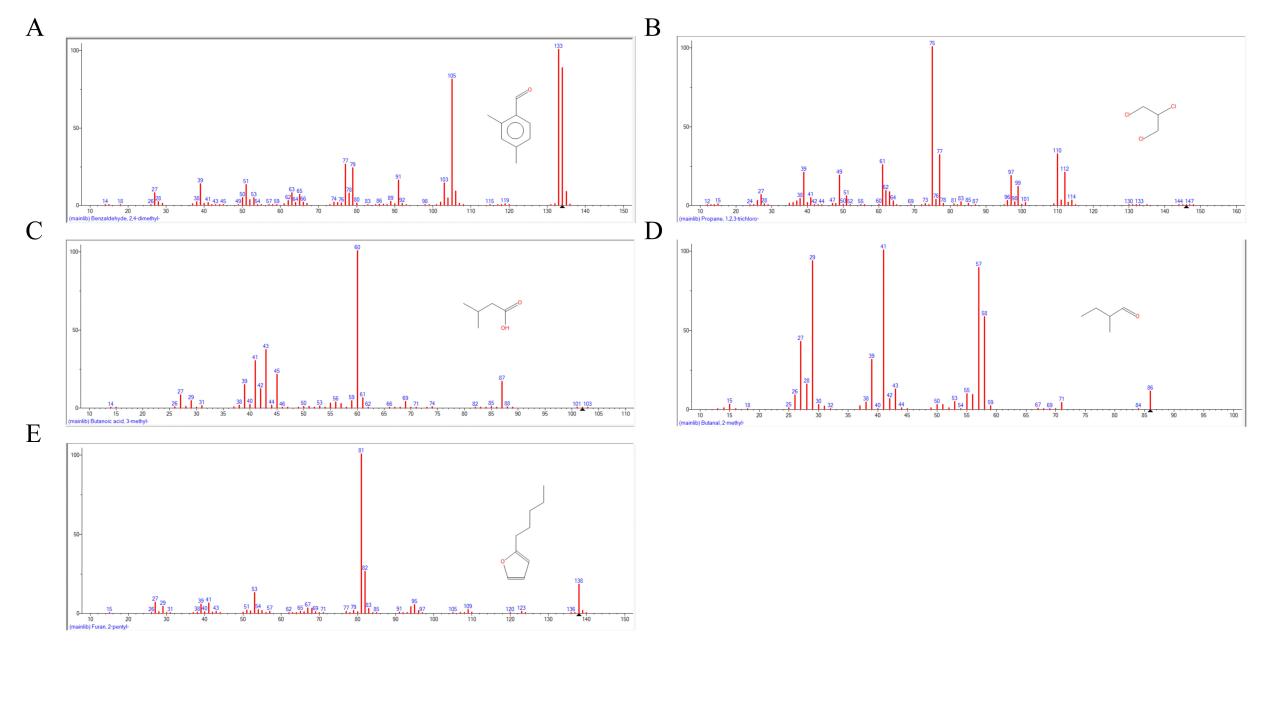


**Supplementary Figure S2:** Volatile component identification mass spectrum: (A) 2,4-dimethyl benzaldehyde, (B) 1,2,3-trichloropropane, (C) isovaleric acid, (D) 2-methylbutyraldehyde, (E) 2-amylfuran.


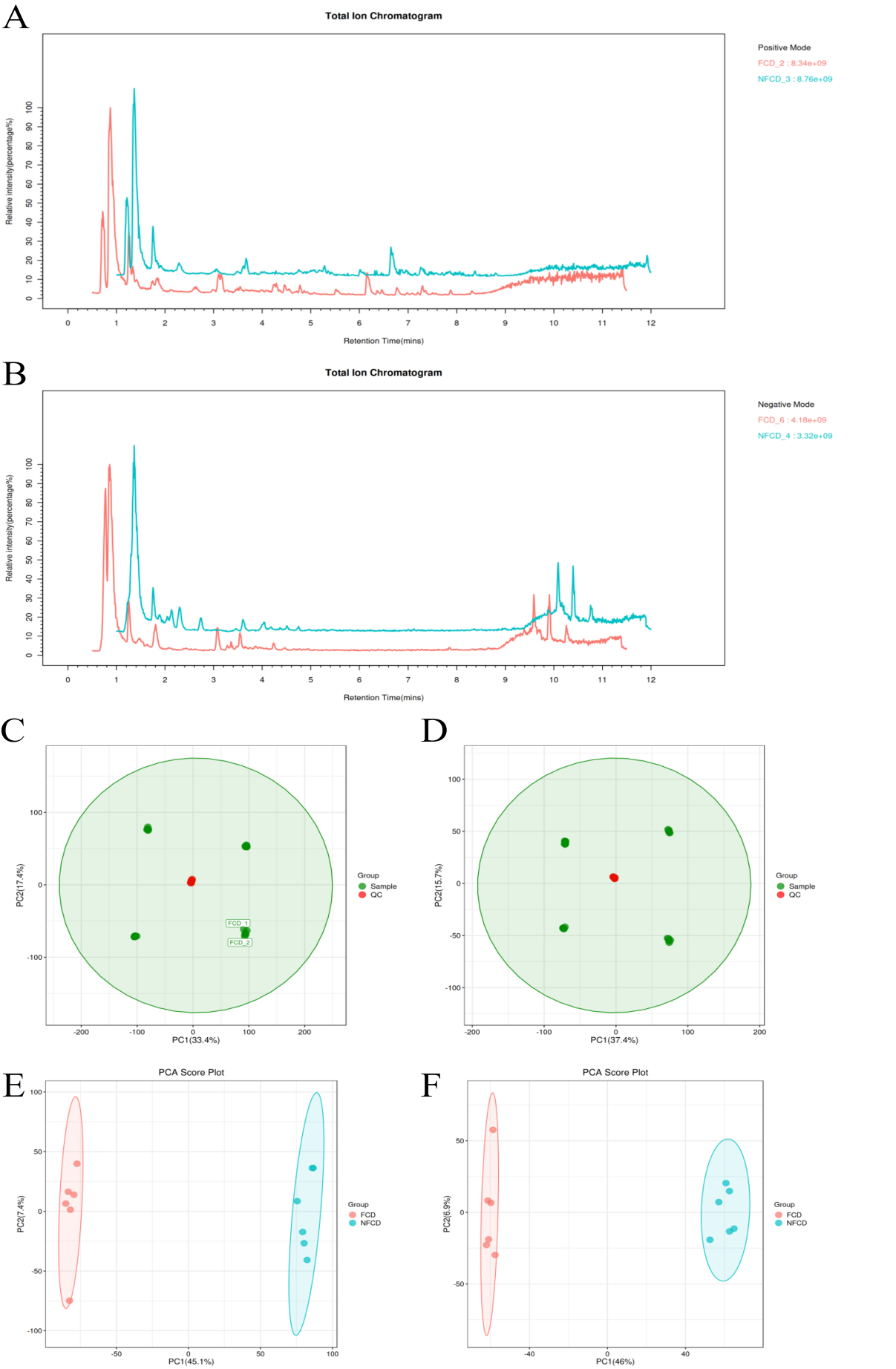


**Supplementary Figure S3.** (A) and (B), Total ion chromatogram (TIC) map; (C) and (D) Quality Control (QC) map; (E) and (F) PCA score plots. (A), (C) and (E) were detected in the positive ion mode; (B), (D) and (F) were detected in the negative ion mode. The abscissa is the retention time of the metabolites detected.


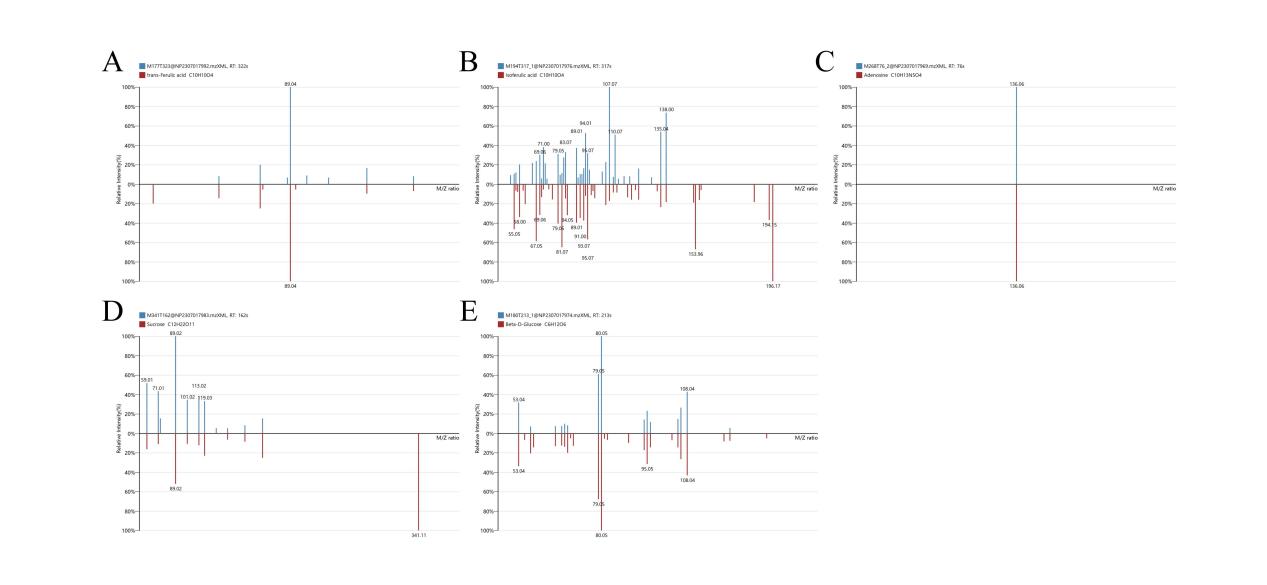


**Supplementary Figure S4:** Mass spectral identification of 5 differential metabolites related to energy metabolism: (A) trans-Ferulic acid, (B) Isoferulic acid, (C) Adenosine, (D) Sucrose, (E) Beta-D-Glucose.
